# Additive Effect on the Structure of PEDOT:PSS Dispersions and Its Correlation with the Structure and Morphology of Thin Films

**DOI:** 10.3390/polym14010141

**Published:** 2021-12-30

**Authors:** Edgar Gutierrez-Fernandez, Tiberio A. Ezquerra, Mari-Cruz García-Gutiérrez

**Affiliations:** Instituto de Estructura de la Materia (IEM-CSIC), Serrano 121, 28006 Madrid, Spain; t.ezquerra@csic.es

**Keywords:** PEDOT:PSS, additive, co-solvent, secondary doping, SAXS, thin films

## Abstract

We reported on the interaction between poly(3,4-ethylenedioxythiophene):polystyrene sulfonate (PEDOT:PSS) and high-boiling-point additives in PEDOT:PSS aqueous dispersions and in the final polymer films with the aim of stablishing correlations between the structure of both inks and solid thin films. By Small-Angle X-ray Scattering (SAXS) using synchrotron radiation, it was found that the structural changes of dispersions of PEDOT:PSS with high-boiling-point additives can be explained as a two-step mechanism depending on the additive concentration. A compaction of PEDOT:PSS grains was observed at low concentrations while a swelling of the grains together with a phase segregation between PEDOT and PSS segments was evidenced at larger concentrations. Thin films’ morphology and structure were investigated by atomic force microscopy (AFM) and synchrotron Grazing Incidence Wide-Angle X-ray Scattering (GIWAXS) respectively. Our two-step model provides an explanation for the small and sharp domains of PEDOT:PSS thin films observed for low-additive concentrations (first step) and larger domains and roughness found for higher-additive concentrations (second step). A reduction of the ratio of PSS in PEDOT:PSS thin films upon the presence of additives was also observed. This can be related to a thinning of the PSS shells of PEDOT:PSS grains in the dispersion. The results discussed in this work provide the basis for a controlled tuning of PEDOT:PSS thin films structure and the subsequent electrical properties.

## 1. Introduction

Extensive research has been performed in order to develop electrically conductive polymer materials, either by direct synthesis of conjugated polymers [1] or by the incorporation of conductive nanoadditives into the insulating polymer matrix [2,3]. Among conjugated polymers, poly(3,4-ethylenedioxythiophene):poly(styrenesulfonate) (PEDOT:PSS) is especially interesting because it is dispersed in water, highly conductive, and almost transparent in the visible region when processed as thin film. In addition, it presents high stability at ambient conditions [4]. PEDOT:PSS is formed by the stable, conjugated polymer poly(3,4-ethylenedioxythiophene) (PEDOT) which is insoluble in water or in any organic solvent and polystyrene sulfonate (PSS), which is water-soluble and acts as a charge-balancing counter ion. PEDOT:PSS is typically available in aqueous dispersion promoting the formation of micelles. The hydrophobic polycationic PEDOT segments tend to form a core of dispersed grains surrounded by the polyanionic PSS chains, which constitute the hydrophilic shell [4].

Many applications have been accomplished with this polymer. In organic solar cells, PEDOT:PSS is prominent as the hole-extracting layer, enhancing the work function in comparison with the usual transparent electrode indium tin oxide (ITO) and improving the contact between the electrode and the active layer [5]. It is also used in perovskite solar cells [6], OLEDs [7], OFETs [8], and thermoelectrics [9]. Different oxidation levels lead to changes in the visible absorption spectra of PEDOT:PSS, making it suitable for electrochromic windows [10]. Besides its high conductivity, PEDOT:PSS is also a pseudocapacitive material, which makes it interesting as a supercapacitor [11]. Controlled stimuli of PEDOT:PSS films to ambient water opens also the possibility of fabricating humidity sensors [12]. Furthermore, its water solubility offers potential applications in biology and medicine [13,14]. However, some of the potential applications for PEDOT:PSS as transparent conducting electrodes require much higher values of electrical conductivity than that provided by the as-received PEDOT:PSS. It is envisioned as a versatile material due to the vast catalogue of physical approaches that has been proved to improve its electrical properties [15]. The incorporation of both liquid [6,16,17,18] or solid additives [19] into the original water dispersion is indeed a simple and effective approach to enhance the electrical conductivity of the material. Other groups showed that carbon nanofillers improve the thermal stability [20] of PEDOT:PSS, and that the hydrogen-bonding interaction between the matrix and a polar filler could be beneficial as well for this purpose [21]. It is clear that the properties of the final device will depend on the interactions between PEDOT:PSS and the additive: (i) in the dispersion; (ii) during the deposition step; and (iii) in the final post-deposition treatment [22,23]. The full interplay between PEDOT:PSS and liquid additives during the whole procedure is not clearly understood. This work is aimed to clarify the influence of polar additives with a high boiling point into the morphology of dispersed PEDOT:PSS and its subsequent solid-state thin film.

At this moment, the effect of the additive, commonly referred to as “secondary doping”, has been explained in terms of its solubility with both the anions of PSS and the neutral segments of polystyrene sulfonic acid (PSSH) [24,25]. This effect leads to conformational changes [26] and crystallinity enhancement of PEDOT oligomers [27,28]. All these effects have been correlated with a phase segregation between segments of PEDOT and PSS, initially strongly bounded by electrostatic forces [29]. Transparent and conducting supported thin films can be prepared from PEDOT:PSS inks [30,31,32]. Free-standing films can also be prepared by using different deposition procedures including solution cast [33], spin-coating [34,35], electro-spinning [36], or inkjet printing [23,27,37], among others. In all cases, the final properties may strongly depend on film uniformity [38,39], on the thickness [40,41], and roughness [39]. As previously mentioned, several works have focused on the study of properties of deposited thin films of PEDOT:PSS treated with polar solvents [16,28,42,43,44,45]. The interaction between PEDOT:PSS and polar additives in the aqueous dispersion and in the final thin films has also received some attention. However, only a few reports [46] focused on the correlations between aqueous suspensions and thin films morphology. Thus, it is pertinent to unveil the influence of a modified ink of PEDOT:PSS on the morphology of the deposited thin film in order to develop protocols that will improve the final properties.

In this context, the present work focused on the effect of ethylene glycol (EG), glycerol (G), dimethyl sulfoxide (DMSO), and the blend (G+DMSO) on: (i) the structure of PEDOT:PSS aqueous dispersions and (ii) the structure and morphology of thin films. The structure of the PEDOT:PSS inks was investigated by small-angle X-ray scattering (SAXS) using synchrotron radiation. The results were discussed in terms of different models. Thin films morphology and structure were investigated by atomic force microscopy (AFM) and by synchrotron grazing incidence wide-angle X-ray scattering (GIWAXS) respectively. Finally, correlations between the structure of thin films and inks were established.

## 2. Materials and Methods

PEDOT:PSS aqueous dispersion (Heraeus Clevios™ AI 4083) was purchased from Ossila Ltd, Sheffield, UK. The concentration of PEDOT:PSS was 1.3% by weight and the PEDOT:PSS ratio was 1:6. Other chemicals, including ethylene glycol (EG), glycerol (G), and dimethyl sulfoxide (DMSO) were purchased from Sigma-Aldrich (Madrid, Spain) and used as received. The chemical structures of all materials are shown in the Supplementary Information (Figure S1). The PEDOT:PSS aqueous dispersion was filtered through a 0.2 µm cellulose filter and different additives quantities (in wt%) were added into the PEDOT:PSS dispersion (Table 1) and finally sonicated for 5 min. For the characterization of samples by SAXS in transmission geometry, the dispersions were poured in glass capillaries (Hilgenberg, Malsfeld, Germany, diameter = 1.5 mm, wall thickness = 0.01 mm) and then hermetically sealed with hot glue in order to avoid evaporation during the measurements.

Thin films were prepared by spin-coating. Squares of n-silicon wafers (100, Arsenic dopant, Neyco, Vanves, France (France)) were used as substrates, which were sonicated in acetone for 10 min. Subsequently, they were cleaned with isopropyl alcohol for another 10 min and finally rinsed in deionized water. Then, the substrates were dried with nitrogen blow. A spin-coating equipment (Laurell WS-650 Series by Laurell, North Wales, PA, USA) was used to prepare the thin films. A fixed amount of 0.1 mL of the dispersion was dropped by a pipette on the substrate and then a rotation rate of 3000 rpm was applied for 60 s. The films were thermally annealed at T = 140 °C for 10 min to remove the additives from the films.

Small-Angle X-ray Scattering (SAXS) experiments were performed at room temperature, at the NCD-SWEET beamline at ALBA synchrotron (Cerdanyola del Vallès, Barcelona, Spain). The X-ray beam with a wavelength *λ* = 0.1 nm (E = 12.4 keV) impinged the sample in transmission geometry, and the SAXS signal was recorded by a Pilatus 1M detector located at 2.191 m from the sample position. Sample-to-detector distance and reciprocal space were calibrated using Silver Behenate. The scattering data were reduced by azimuthal integration of the isotropic 2D SAXS patterns through the whole *q* range, with *q* being the modulus of the scattering vector (*q* = *4π*/*λ*(sin*θ*), where *2θ* is the scattering angle). The resulting intensity profiles were normalized in terms of photon current and absorbance of the sample, and finally corrected by subtracting the profile from a pure water sample.

Grazing-Incidence Wide-Angle X-ray Scattering experiments (GIWAXS) were performed at ALBA synchrotron, BL-11 (NCD-SWEET). The X-ray beam wavelength was set at *λ* = 0.1 nm. GIWAXS patterns were collected by a LX255-HS 2D (Rayonix) area detector, placed at 11 cm from the sample. Set-up parameters were calibrated using Cr_2_O_3_.

Atomic Force Microscopy (AFM) measurements were performed with a scanning probe microscope (MultiMode 8 equipped with a Nanoscope V controller, Bruker, Kasruhe, Germany). Topography images were collected in tapping mode using silicon probes (Tap 300GB-G probes by BudgetSensors, Sofia, Bulgaria). The thickness of the films was obtained measuring the height of a scratch. The grain size analysis from the AFM images was performed with ImageJ software using the particles analysis plugin.

Dynamic Light Scattering (DLS) experiments were performed with a Malvern Zetasizer Nano SZ (Malvern Panalytical, Malvern, UK) equipment using disposable folded cuvettes. The PEDOT:PSS dispersion was filtered with a PVDF 200 nm syringe filter and diluted thirty times.

## 3. Results and Discussion

**Figure 1:**
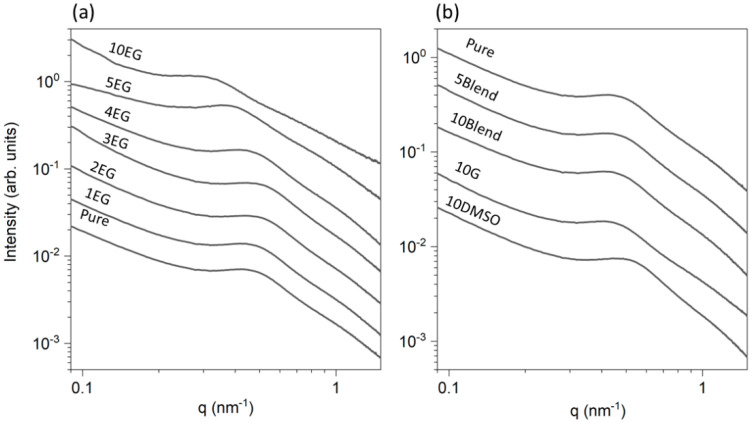
SAXS intensity profiles of PEDOT:PSS (**a**) with EG and (**b**) with G, with DMSO and the blend G+DMSO, at different wt% as labeled. X-axis and Y-axis are presented in logarithmic scale. The profiles are shifted vertically for clarity.

The diameter of the PEDOT:PSS particles within the aqueous dispersion was first measured by Dynamic Light Scattering experiments (DLS). The results indicated that the dispersion was formed by particles with diameters between 20 nn and 200 nm whose size distribution was centered at 50 nm (Figure S2). These values are in agreement with other works [47]. It has been suggested that the dispersion stability of PEDOT:PSS is provided by a high concentration of negatively charged PSS monomers in the outer region of the particles, promoting electrostatic repulsion among particles preventing aggregation. Figure 1 presents the intensity profiles as a function of the scattering vector *q* for the different samples.

The SAXS profiles from PEDOT:PSS dispersions can be separated in different contributions, depending on the *q*-range [48]: (i) two negative slopes, at low *q* (~0.1 nm^−1^) and (ii) another slope at high *q* (~1 nm^−1^). This last region is associated with distances around 60 nm and 6 nm respectively. In addition, a well-resolved maximum was located at *q* ~ 0.4 nm^−1^ (*d*~15 nm). The component at low *q* originated from the aggregation of colloidal grains, whereas the maximum arose from a correlation length within the grains. The maximum was located at similar *q* values for all the samples. Thus, we consider that the correlation distance can be associated to a characteristic length within the PEDOT:PSS grains, being that these grains were formed by a PEDOT-rich core and a PSS-rich shell. It has been previously reported that this maximum is related to the well-known polyelectrolyte peak which arises from rod-like segments along the chains as a consequence of the electrostatic repulsion of the highly charged backbone [42,49]. A recent work reported that this maximum is associated with the form factor of the core-shell structure [46]. At a first glance, a shift of the peak maximum to either lower or higher *q* values with respect to the pristine dispersion, depending on the EG concentration, was observed and is shown in Figure 1a. This effect was less evident in the case of G and DMSO (Figure 1b). The asymptotic behavior of the SAXS intensity at high *q*-values defines what is called the Porod regime. In this regime, the SAXS intensity scattered by a mass fractal follows Porod’s law:

(1) $$I\propto q^{-d_{m}}$$

where *d_m_* is the fractal dimensionality of the scatterer [50,51]. For a one-dimensional randomly oriented rod-like scatterer, *d_m_* = 1, while disk-like objects or a Gaussian random coiled chain display *d_m_* = 2. For three-dimensional objects, Equation (1) is not valid since the scattering from the surface of the object has to be taken into account. In these cases, the surface is characterized by a surface fractal dimension, *d_s_*, which relates the surface area to the size of the scattering object. For these cases, Equation (1) can be generalized as being:

(2) $$I\propto q^{-\left(2d_{m}-d_{s}\right)}$$

For a sharp, non-fractal surface of a three dimensional object, *d_s_* = 2 and *d_m_* = 3; thus, Porod’s Law becomes *I* ∝ *q*^−4^, while less negative exponents were found for rougher, fractal surfaces. The intensity profiles were fitted taking the same *q*-range for all the samples (0.7 < *q*/nm^−1^ < 1.1). The exponents are listed in Table 2.

The intensity profile from a pure PEDOT:PSS dispersion decays as *I* ∝ *q*^−1.91^, suggesting a random coil chain conformation. Above 4 wt.%EG concentration, the exponent reduces considerably, suggesting a clear modification of the structure from a random coil to a rod-like scatterer. A similar interpretation can explain the electrostatic interaction between PEDOT:PSS and ionic dopants [46]. On the other hand, while G induced a similar effect as EG at the same concentration, DMSO seemed to induce the opposite, i.e., a more negative exponent.

**Figure 2:**
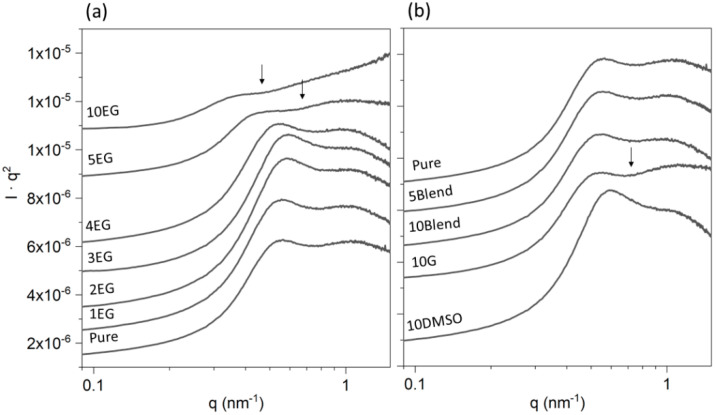
Kratky plots calculated from the SAXS intensity profiles of PEDOT:PSS with EG (**a**) and with G, with DMSO and G+DMSO (**b**), at different wt.% as labelled. The arrows highlight the onset for the upturn of intensity. X-axis are shown in logarithmic scale. The profiles are shifted vertically for clarity.

In order to better visualize the impact of additives on the power decay, we used Kratky plots (*I*(*q*)·*q*^2^ vs. *q*) [52] (Figure 2). Kratky plots are commonly used in the analysis of protein solution SAXS to assess the flexibility and shape of the sample. In this case, an absence of slope at high *q*-values can be straightforwardly interpreted as being the characteristics of a random coil Gaussian chain.

The Kratky plot of the pristine dispersion (Figure 2a,b) clearly exhibits the maximum around *q* ~ 0.4 nm^−1^ associated with the polyelectrolyte nature of the sample, followed by a small plateau and a decay for *q* > 1 nm^−1^. The typical decay of a random coil structure (*I* ∝ *q*^−2^) should turn into a plateau in the Kratky plot [53].

At 5 wt.% and higher EG concentrations, the plateau upturns for *q* > 0.7 nm^−1^. This effect indicates an intensity decay exponent lower than 2, which can be explained in terms of the persistent chain model [54]. According to this model, for a certain length scale, consecutive chain segments bend with small angles forcing the chain to exhibit a continuous curvature. The relative rigidity of the chain can be quantified by the persistence length. Therefore, if the scattering object is close to a rigid rod, at high *q*-values, the intensity decays by a lower exponent (Equation (1)). In this case, the SAXS profiles should exhibit a transition *q*-value, q* (arrows in Figure 2) between the plateau and the upturn. The q* value is related to the persistence length, *a*, by the following expression [54]:

(3) $$a=\frac{1.91}{q^{*}}$$

For the PEDOT:PSS dispersion with 5 and 10 wt% EG (Figure 2a), the q* values can be estimated as being around 0.62 nm^−1^ and 0.42 nm^−1^ respectively. These values correspond to persistence lengths of *a*~3 nm and 4.5 nm, respectively. The SAXS profile of the sample with 10 wt.% G (Figure 2b) also showed an upturn for *q >* 0.75 nm^−1^ (*a*~2.5 nm). Interestingly enough, DMSO (Figure 2b) seemed to induce the opposite effect than EG and G, compensating the effect of G in the samples with the blend G+DMSO.

From the Kratky plot analysis, we can conclude that EG does not induce a significant effect on the structure of aqueous dispersion of PEDOT:PSS until a concentration of 4 wt% is reached. At this value, PEDOT:PSS chains, which initially arrange according to a random coil configuration, gradually stiffen and behave as rod-like, semi-rigid entities. This effect is reinforced by increasing the EG concentration. The stiffening of the chains is likely to be induced upon the interaction of EG through hydrogen bonding between charged PEDOT and PSS. The polar additive (EG) screens the Coulombic interaction between charged segments which adopt less constrained and more planar conformations. This effect improves the conductivity in PEDOT:PSS thin films [26,29]. While G seems to induce the same effect on the PEDOT:PSS aqueous dispersion, DMSO, by contrast, induces more compact structures. It is noteworthy that EG and G have a similar relative polarity value (0.79 and 0.81 respectively). These values are higher than that from DMSO (0.44). Thus, it is expected that a concentration of 10 wt% DMSO has a comparable effect as lower concentrations of EG since DMSO induces a weaker interaction with PEDOT:PSS.

**Figure 3:**
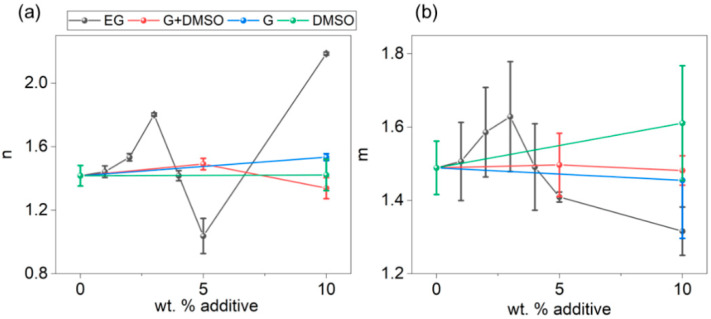
(**a**) Low-*q* exponent *n* and (**b**) high-*q* exponent *m* of PEDOT:PSS dispersions with different concentrations of EG (black points), G (blue points), DMSO (green points), and the blend (G+DMSO) (red points).

In order to establish quantitative trends upon the incorporation of the additives within the dispersion, the intensity profiles were fitted in the whole *q*-range (0.08–1.5 nm^−1^) according to an empiric ‘broad peak’ model [49]. This model has been applied to electrolytic systems [8]. In this case the SAXS intensity is described by:

(4) $$I\left(q\right)=\frac{A_{0}}{q^{n}}+\frac{A_{1}}{1+{\left(L\cdot \left|q-q_{max}\right|\right)}^{m}}+A_{2}$$

where *A*_0_, *A*_1_, and *A*_2_ are scaling factors, *n* and *m* are the low-*q* and high-*q* scaling exponents, respectively, and *L* describes the screening length of the inter-chain interaction [49]. Both *n* and *m* exponents describe the dimensionality of the scattering object at different distances. While *n* is relevant for distances of tens of nanometers, which is of the order of the PEDOT:PSS grain size, *m* is for distances around 1–10 nm corresponding to the length scale of the inter-chain interactions [49]. The peak, *q_max_*, as mentioned before, can be related to a correlation distance *ξ* = 2π/*q_max_* between charged chains due to electrostatic repulsions. All the intensity SAXS profiles can be reasonably fitted to the experimental ones (Figure S3). If PEDOT:PSS grains are visualized as cores of PEDOT with a shell of PSS, then *ξ* can be interpreted as an average distance between the center of the core (positive region) and the middle of the PSS shell (negative region). Figure 3 shows *n* and *m* values obtained from the fitting for PEDOT:PSS dispersions with different concentrations of EG, G, DMSO, and G+DMSO.

The low-*q* exponents from dispersions of PEDOT:PSS with EG (Figure 3a, black points) tended to increase in most of the concentrations with respect to the pristine value. The *n* values corresponding to 5 wt% and 10 wt% EG were less accurate due to the broadening of the peak in SAXS intensity profiles (Figure 1a) affecting the slope from the aggregates at this *q*-range. The *n* values for G, DMSO, and the blend were similar in the analyzed range. Concerning the high-*q* exponent region, a similar trend was observed (Table 2).

The increase of *n* at low EG concentrations can be associated with the presence of denser aggregates in the dispersions. As above discussed, a reduction of *m* can be attributed to a transition from a coil-like chain conformation to a more rigid one in the dispersion [49,55]. The fitted data from dispersions with EG and G support this behavior. These results are in line with those extracted from Kratky plots concerning the addition of EG to the PEDOT:PSS aqueous dispersion (Figure 2a). A similar conclusion can be suggested for PEDOT:PSS dispersion with G, supporting that the differences can be associated with the high polarity of G. This solvent should interact more efficiently with the charged segments of PSS and PEDOT [55]. Thus, the screening of the Coulombic force would induce a more-extended chain conformation. On the contrary, DMSO seems to affect the PEDOT:PSS dispersion at both the interchain and nanometer distance level as derived from Kratky plots analysis (Figure 2b) but not remarkably affecting the PEDOT:PSS grain structure.

**Figure 4:**
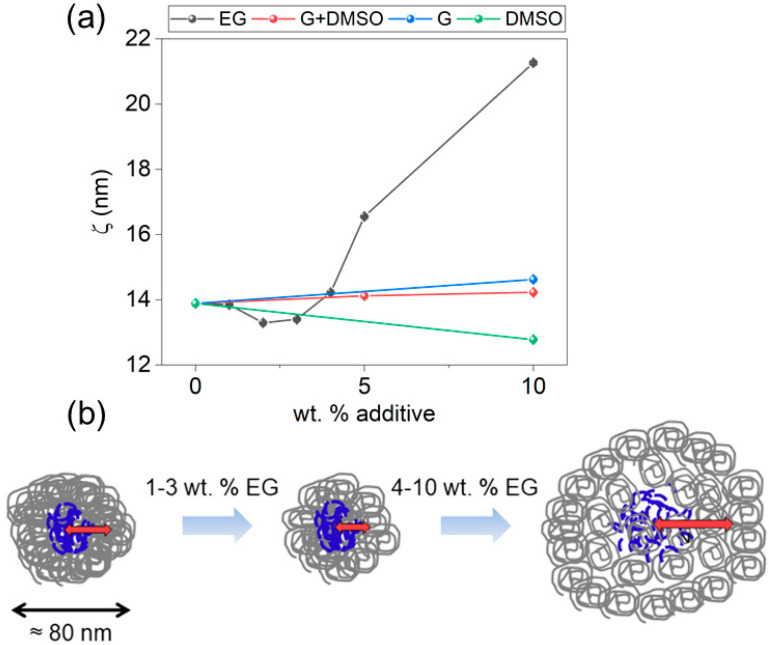
(**a**) Correlation length *ξ* for PEDOT:PSS grains at different concentrations of EG (black points), G (blue points), DMSO (green points), and the blend (G+DMSO) (red points). (**b**) Representation of the effect of EG on the PEDOT:PSS grains within the dispersion. Red arrows represent the idea behind correlation length *ξ*. Scheme not in scale.

The obtained values for the screening length of the inter-chain interaction, *L ≈* 4.5 ± 0.5 nm, were similar for all systems. Regarding the correlation distance, *ξ* = 2π/*q_max_*, the trends for every additive concentration are presented in Figure 4a.

Clearly, EG had a higher interaction with *ξ* than the other additives. In the case of EG (Figure 4a, black points), *ξ* dropped slightly from *ξ* = 14 nm at low concentrations (for 1–3 wt%) and increased at higher concentrations, reaching a maximum of *ξ* = 21 nm. For PEDOT:PSS dispersion with 10 wt% of G (Figure 4a, blue points), *ξ* slightly increased compared with that of the pristine value. In the case of 10 wt% of DMSO (Figure 4a, green points), *ξ* decreased. The results from dispersions with blend G+DMSO (Figure 4a, red points) presented a slight increase with higher concentrations, showing an intermediate situation between the PEDOT:PSS dispersions with G and DMSO. We can explain the reduction of *ξ* at low EG concentrations as due to a compaction of the PEDOT:PSS grains, as observed in other polylelectrolyte systems [56]. It is reported that spin-coated thin films of PEDOT:PSS with EG contain less PSS amount than pristine PEDOT:PSS thin films because the EG interacts with the excess of PSS, which is ejected during the spin step [57]. Thus, at low concentrations of additive, EG interacts with the PSS shells of PEDOT:PSS grains, removing it partially. The grains would suffer a stronger Coulombic interaction between unbalanced charged regions that would collapse the structure. This would reduce the correlation length between charged zones *ξ* (Figure 4a, black points) and also could explain the densification of the larger aggregates, as revealed by the increase of the low-*q* exponent in Figure 3a (black points).

The increase of *ξ* above 4 wt% EG points toward the swelling of the PEDOT:PSS grains. We explained the two-stage effect of EG considering the scheme of Figure 4b. At concentrations ≥4 wt%, the additive interacts with charged PEDOT and PSS inducing more extended chains and a swelling of the grains in the order of tens of nanometers. This model is coherent with our conclusions based on the SAXS data in the frame of the Kratky plots analysis (Figure 2a, black data). This model is coherent with reported DLS results showing larger PEDOT:PSS grains when the dispersion is mixed with poly(ethylene glycol) [58].

In the case of G, it seemed to follow a similar behavior as EG. However, the effect was less pronounced (Figure 4a, blue data). In the case of DMSO, the trend of *ξ* exhibited a slight decrease upon the addition of 10 wt% DMSO. For similar concentrations, Murphy et al. reported almost no change in the SAXS profile, and Bagchi et al. reported an increase of *ξ* but for much larger concentrations of DMSO.

### Additive Effect on the Morphology and Structure of PEDOT:PSS Thin Films

**Figure 5:**
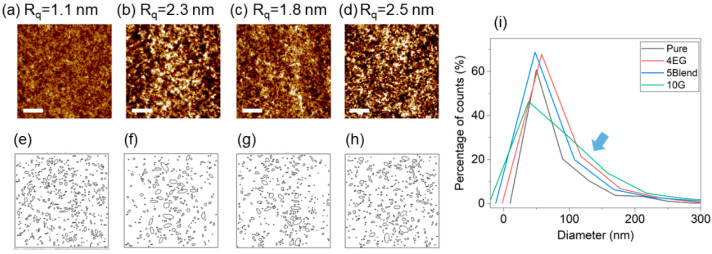
(**a**–**d**) AFM topographical images obtained in tapping mode with their corresponding roughness value: (**a**) pristine PEDOT:PSS, (**b**) PEDOT:PSS with 4 wt% EG, (**c**) PEDOT:PSS with 5 wt% (G+DMSO) and (**d**) PEDOT:PSS with 10 wt% G. (**e**–**h**) AFM height images after particle analysis processing. (**i**) Distribution of grain diameters analyzed by particle analysis plug-in of ImageJ software, version 1.53e.

Thin films from the investigated dispersions were prepared by spin-coating. It was found that DMSO dispersions and EG dispersions with concentrations > 4 wt% did not form continuous films under the used spin-coating conditions. Figure 5 shows AFM topographical images of pure PEDOT:PSS thin films and mixed with the additives.

The additive induced rougher surfaces from a value R_q_~1 nm for a pure PEDOT:PSS thin film to systematically higher values for the different additive-assisted films around R_q_~2–2.5 nm (Figure 5a–d). The increase of roughness in PEDOT:PSS films doped with EG can be attributed to either thinning or removal of PSS segments within PEDOT:PSS grains [59,60,61]. However, looking at the morphology of the AFM images, it is possible to elucidate that the surface consisted of grain-like domains (Figure 5e–h). Independently of the additive, an enhancement of the grain sizes was observed. PEDOT:PSS exhibited a distribution of grain sizes centered on 50 nm, in agreement with the DLS experiments (Figure S2). Thin films of PEDOT:PSS with additives exhibited broader and polydisperse distributions ranging from 50 to 200 nm in diameter (highlighted with a blue arrow in Figure 5i).

**Figure 6:**
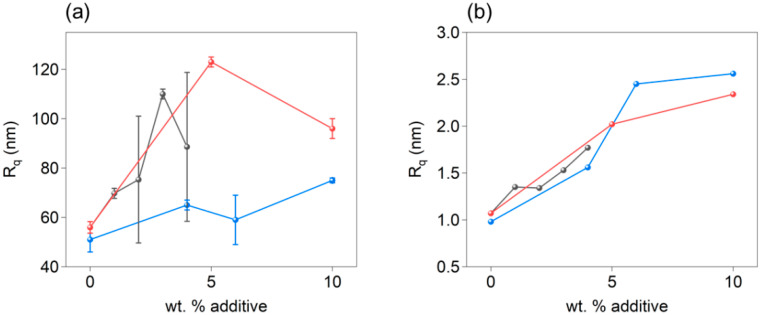
(**a**) Thickness and (**b**) roughness (R_q_) of thin films of PEDOT:PSS with EG (black data), G (blue data), and G+DMSO (red data).

Smaller domains have been measured by AFM in thicker PEDOT:PSS films with EG [62]. In this case, a reduction of PSS shells during spin-coating is expected. However, enlargement of PEDOT:PSS grains has also been identified by AFM on spin-coated thin films [63]. This effect has been attributed to the coalescence of PEDOT:PSS grains with lower amount of PSS. This apparent contradiction can be understood taking into account that different thermal annealing treatments lead to the coalescence of the grains and the formation of larger domain sizes. The larger grains detected upon mixing with G and G+DMSO may be explained by the swelling of PEDOT:PSS grains after addition of higher additive concentrations, as explained previously. Figure 6 shows the thickness and roughness dependence of PEDOT:PSS thin films as a function of additive concentrations.

The thickness of pure PEDOT:PSS thin films is roughly 50 nm. The incorporation of EG increased the thickness, although the film became irregular, as reflected by the size of the error bars. Higher concentrations than 4 wt% formed discontinuous films. When blended with G or G+DMSO, the thickness also increased as a general trend, although it decreased at 10 wt% of G+DMSO. As previously mentioned, the higher the additive concentration, the rougher the surface (Figure 6b).

The enhanced thickness after deposition and thermal annealing suggest an irreversible reorganization of the material upon the effect of the high-boiling point additive during the deposition. Thermal annealing removes the additive but its effect on the dispersion is retained. In the case of EG, our previous interpretation as a two-stage mechanism can explain why sharper and slightly larger grains were observed in this study and why larger globules are observed by other groups [63]. On the one hand, densification of grains at low EG concentrations provides rougher and granulated surfaces. On the other hand, the swelling detected by SAXS at larger concentrations support larger globules, referred to the structures detected by AFM, around 50–100 nm. In the previous section, larger correlation distances *ξ* were associated with the swelling of PEDOT:PSS grains. Although continuous thin films of PEDOT:PSS with high EG concentrations were not formed, the swelling induced by G can be related to the larger domains detected by AFM (Figure 5). This hypothesis can also be applied for thin films of PEDOT:PSS with G+DMSO.

**Figure 7:**
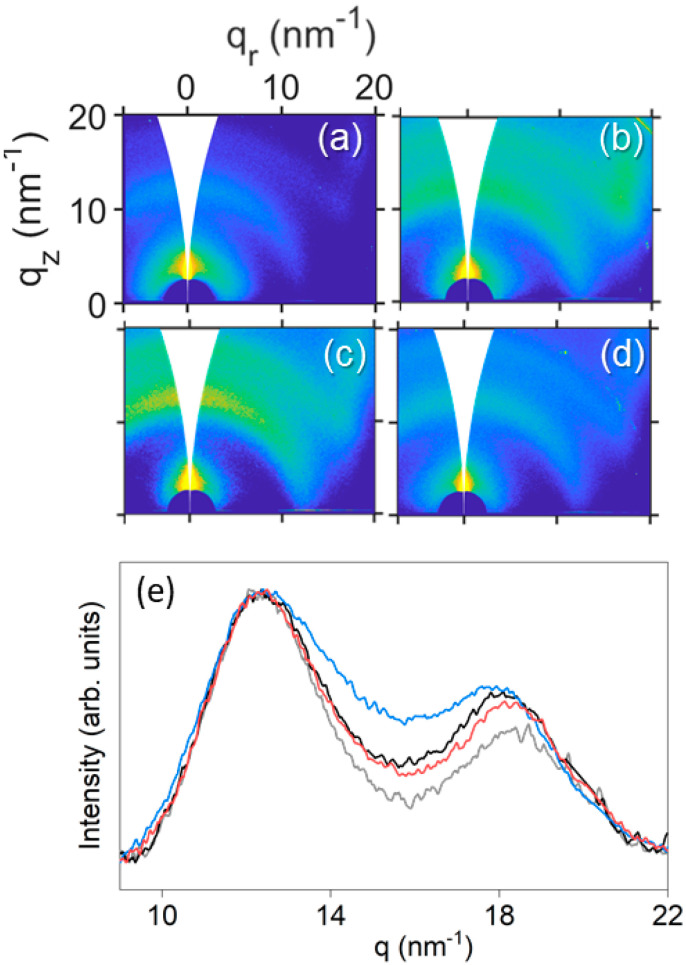
2D GIWAXS patterns from PEDOT:PSS thin films: (**a**) pure, (**b**) 4EG, (**c**) 10G, and (**d**) 10G+DMSO. (**e**) Azimuthal integrations of 2D GIWAXS patterns from pure PEDOT:PSS (gray curve), 4EG (black curve), 10G (blue curve), 10GDMSO (red curve).

The additive effects on the structure of PEDOT:PSS thin films were elucidated by GIWAXS. Figure 7a–d shows GIWAXS patterns from selected samples. The pattern of pristine PEDOT:PSS (Figure 7a) and with additives (Figure 7b–d) presented the same reflections at *q* = 12.5 nm^−1^ and *q* = 18.2 nm^−1^. The first one comes from amorphous PSS, although other works refer to the π-π stacking of PSS [27]. The one at *q* = 18.2 nm^−1^ is associated with the π-π stacking of PEDOT segments [27].

No evidence of re-orientation and peak shifting were found, as those reported by Dong et al. [28]. However, we identified relative changes between both Bragg peaks of PSS and PEDOT. We compared the ratios between the peak area associated with PSS and PEDOT. Other groups have seen a PSS reduction in the surface of films upon the interaction of the additive and PSS [29,57]. In this work we observed an intensity decrease on the PSS Bragg reflection. In order to compare patterns from different samples in an accurate way, the intensity profiles were corrected from background scattering and normalized to the maximum of PSS peaks (Figure 7e). The PEDOT and PSS contributions in the intensity profiles were fitted to Voigt functions and the relation between their areas (A_PSS_/A_PEDOT_) are included in Table 3.

The ratio of PSS in PEDOT:PSS thin films with the investigated additives was lower than in pristine PEDOT:PSS thin films, being the lowest for EG even with lower concentration. This intensity reduction supports the partial removal of PSS during spin-coating due to interaction of PEDOT:PSS with the additives. As explained for PEDOT:PSS dispersions, the additives induced a phase segregation between PEDOT and PSS, and due to the hydrophilic character of PSS, it was ejected during spin-coating. The reduction of the PSS ratio would improve the conductive properties of PEDOT:PSS thin films [39,48,64].

## 4. Conclusions

High-boiling point additives modify the structure and conformation of PEDOT:PSS water dispersion following a two-step mechanism, depending on additive concentration. A compaction of PEDOT:PSS grains was observed at low concentrations while a higher amount of the additive induced swelling on the grains, possibly due to screening between charged segments of PSS and PEDOT. Thin films formed by the investigated dispersions exhibited a trend of increasing film thickness and roughness with increasing additive concentration. In addition, a direct correlation between the structure of the PEDOT:PSS dispersions and the morphology and internal structure of the corresponding spin-coated thin films was evidenced. The two-stage mechanism described for the effect of additives on the dispersion structure provides an explanation for the small and sharp domains of PEDOT:PSS observed for low additive concentrations (first step), and larger domains and roughness for higher additive concentrations (second step). Finally, the ratio of PSS in PEDOT:PSS thin films with the investigated additives was lower than in pristine PEDOT:PSS thin films, being the lowest for EG even with lower concentrations.

## Figures and Tables

**Table 1:** Additives, additive boiling point, polarity, and sample name, where wt.% is the weight per cent between the water of the pristine PEDOT:PSS dispersion and the additive.

Additive	Boiling Point (°C)	Polarity in Relation with Water	Sample Name
Ethylene Glycol (EG)	198	0.79	wt% EG
Glycerol (G)	290	0.81	wt% G
Dimethyl Sulfoxide (DMSO)	189	0.44	wt% DMSO
Blend (G+DMSO)	-	-	wt% G+DMSO

**Table 2:** Decay exponent for every SAXS intensity profile in the high-*q* range *q* = 0.7–1.1 nm^−1^.

EG (wt.%)	Exponent	G (wt.%)	Exponent
0	−1.91	10	−1.78
1	−1.96	DMSO (wt%)	
2	−2.05	10	−2.2
3	−2.10	G+DMSO (wt%)	
4	−1.96	5	−1.95
5	−1.78	10	−1.97
10	−1.52		

**Table 3:** Ratio of PSS (A_PSS_/A_PEDOT_) in PEDOT:PSS thin films prepared from dispersions with different additives.

	Pristine	4 wt% EG	10 wt% G	10 wt% G + DMSO
A_PSS_/A_PEDOT_	0.98 ± 0.04	0.79 ± 0.08	0.80 ± 0.01	0.91 ± 0.05

## Data Availability

The data presented in this study are available on request from the corresponding author.

## Supplementary Materials

The following supporting information can be downloaded at: https://www.mdpi.com/article/10.3390/polym14010141/s1. Chemical structures of the polar additives (Figure S1), distribution of particle diameters by DLS (Figure S2) and fitted SAXS profiles from PEDOT:PSS dispersions (Figure S3).
