# Supplementary material for: Additive Effect on the Structure of PEDOT:PSS Dispersions and Its Correlation with the Structure and Morphology of Thin Films
Source: Polymers (Basel). 2021 Dec 30;14(1):141. doi: 10.3390/polym14010141 (PMC8747737; doi:10.3390/polym14010141)
Supplement: Supplementary file 1 [file polymers-14-00141-s001.zip › polymers-1493294-supplementary.pdf]

polymers

Supplementary information

Additive Effect on the Structure of PEDOT:PSS Dispersions and Its Correlation with the Structure and Morphology of Thin Films

Edgar Gutierrez-Fernandez *,†, Tiberio A. Ezquerra and Mari-Cruz García-Gutiérrez *

Instituto de Estructura de la Materia (IEM-CSIC), Serrano 121, 28006 Madrid, Spain; t.ezquerra@csic.es
* Correspondence: edgar.gutierrez@ehu.eus (E.G.-F.); maricruz@iem.cfmac.csic.es (M.-C.G.-G.)
† Present address: POLYMAT, University of the Basque Country UPV/EHU, Av. Tolosa 72, 20018 San Sebastian, Spain.

Citation: Gutierrez-Fernandez, E.; Ezquerra, T.A.; García-Gutiérrez, M.-C. Additive Effect on the Structure of PEDOT:PSS Dispersions and Its Correlation with the Structure and Morphology of Thin Films. *Polymers* **2022**, *14*, 141. https://doi.org/10.3390/polym14010141

Academic Editor: Jorge Escorihuela Fuentes

Received: 18 November 2021
Accepted: 28 December 2021
Published: 30 December 2021

Publisher's Note: MDPI stays neutral with regard to jurisdictional claims in published maps and institutional affiliations.

Copyright: © 2021 by the authors. Licensee MDPI, Basel, Switzerland. This article is an open access article distributed under the terms and conditions of the Creative Commons Attribution (CC BY) license (https://creativecommons.org/licenses/by/4.0/).

1. Supplementary Information

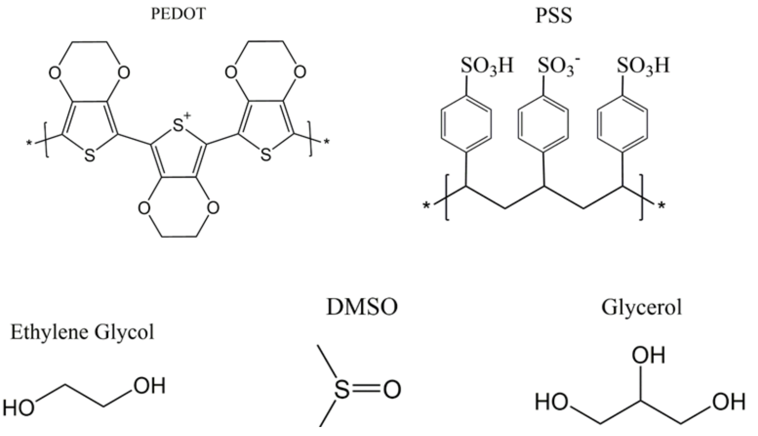

Figure S1. Chemical structure of PEDOT, PSS and the additives ethylene glycol, DMSO and glycerol.

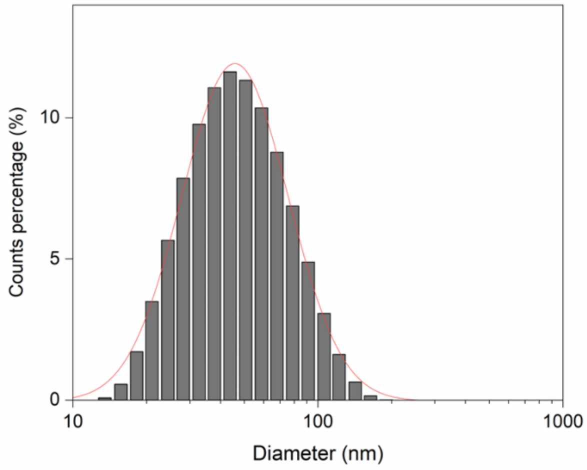

Figure S2. Distribution of particle diameters within a pure PEDOT:PSS dispersion obtained by Dynamic Light Scattering (DLS).

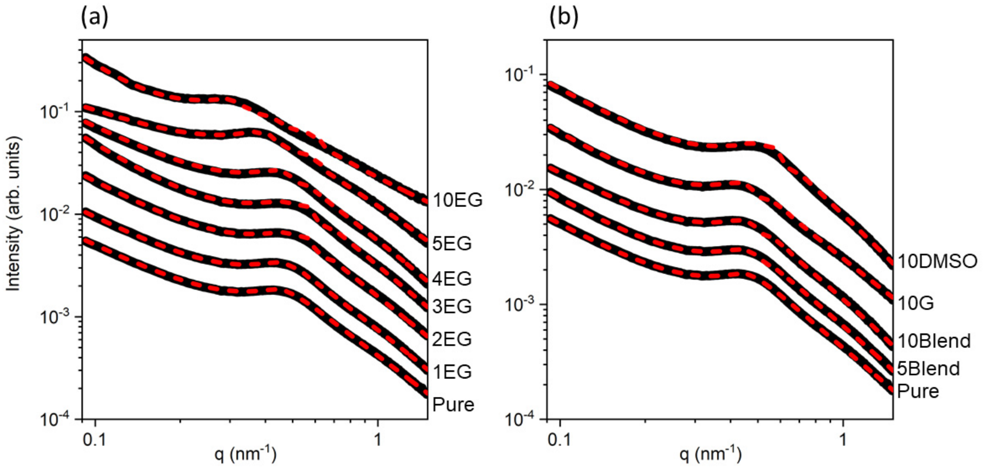

Figure S3. SAXS intensity profile of the PEDOT:PSS dispersions with EG (**a**) and G, DMSO or the blend G+DMSO. (**b**). Black curves represent the SAXS profiles and the red, dashed curves represent the fittings according to Equation 4. X-axis and Y-axis are presented in logarithmic scale.
